# Meta-analysis of variables affecting mouse protection efficacy of whole organism *Brucella *vaccines and vaccine candidates

**DOI:** 10.1186/1471-2105-14-S6-S3

**Published:** 2013-04-17

**Authors:** Thomas E Todd, Omar Tibi, Yu Lin, Samantha Sayers, Denise N Bronner, Zuoshuang Xiang, Yongqun He

**Affiliations:** 1 Unit for Laboratory Animal Medicine, University of Michigan Medical School, Ann Arbor, MI 48109, USA; 2 Division of Comparative Medicine, University of South Florida, Tampa, FL 33612, USA; 3 Department of Biology, Eastern Michigan University, Ypsilanti, MI 48197, USA; 4 Department of Microbiology and Immunology, University of Michigan Medical School, Ann Arbor, MI 48109, USA; 5 Center for Computational Medicine and Bioinformatics, University of Michigan Medical School, Ann Arbor, MI 48109, USA

## Abstract

**Background:**

Vaccine protection investigation includes three processes: vaccination, pathogen challenge, and vaccine protection efficacy assessment. Many variables can affect the results of vaccine protection. *Brucella*, a genus of facultative intracellular bacteria, is the etiologic agent of brucellosis in humans and multiple animal species. Extensive research has been conducted in developing effective live attenuated *Brucella *vaccines. We hypothesized that some variables play a more important role than others in determining vaccine protective efficacy. Using *Brucella *vaccines and vaccine candidates as study models, this hypothesis was tested by meta-analysis of *Brucella *vaccine studies reported in the literature.

**Results:**

Nineteen variables related to vaccine-induced protection of mice against infection with virulent brucellae were selected based on modeling investigation of the vaccine protection processes. The variable "vaccine protection efficacy" was set as a dependent variable while the other eighteen were set as independent variables. Discrete or continuous values were collected from papers for each variable of each data set. In total, 401 experimental groups were manually annotated from 74 peer-reviewed publications containing mouse protection data for live attenuated *Brucella *vaccines or vaccine candidates. Our ANOVA analysis indicated that nine variables contributed significantly (P-value < 0.05) to *Brucella *vaccine protection efficacy: vaccine strain, vaccination host (mouse) strain, vaccination dose, vaccination route, challenge pathogen strain, challenge route, challenge-killing interval, colony forming units (CFUs) in mouse spleen, and CFU reduction compared to control group. The other 10 variables (*e.g*., mouse age, vaccination-challenge interval, and challenge dose) were not found to be statistically significant (P-value > 0.05). The protection level of RB51 was sacrificed when the values of several variables (e.g., vaccination route, vaccine viability, and challenge pathogen strain) change. It is suggestive that it is difficult to protect against aerosol challenge. Somewhat counter-intuitively, our results indicate that intraperitoneal and subcutaneous vaccinations are much more effective to protect against aerosol *Brucella *challenge than intranasal vaccination.

**Conclusions:**

Literature meta-analysis identified variables that significantly contribute to *Brucella *vaccine protection efficacy. The results obtained provide critical information for rational vaccine study design. Literature meta-analysis is generic and can be applied to analyze variables critical for vaccine protection against other infectious diseases.

## Background

Bioinformatics meta-analysis has become an important tool in the analysis of biomedical literature. The term "meta-analysis" was coined by Glass in the 1970s to describe the process of gathering and combining information from many studies of the same type [1]. As a quantitative and systematic process, meta-analysis is able to derive overarching conclusions about a specific area of research. Outcomes from a pooled meta-analysis may include a more precise estimate of the effect of biological factors or outcomes than could be obtained from an individual study. Meta-analysis has the substantial benefit of investigating new hypotheses using existing data [2]. Meta-analysis started to make an impact on medicine in the late 1980s [2,3]. Today, meta-analysis is frequently applied to examine a wide array of different biomedical research areas.

*Brucella *is a genus of facultative intracellular bacteria that are the etiologic agents of brucellosis in humans and many animal species. Brucellosis is the most commonly acquired zoonotic disease, with over half a million new human cases annually worldwide [4]. Vaccination is one of the most effective means to protect humans and animals against brucellosis and other infectious diseases [5]. Although antibodies specific for the O polysaccharide of *Brucella *cell wall lipopolysaccharide confer a certain level of protection in some host species, cell-mediated immunity is believed to play a critical role in protection against virulent *Brucella *infection [6]. As a result, live attenuated *Brucella *vaccines, including RB51 and strain 19 (S19), are superior to many other *Brucella *vaccine candidates as they tend to induce better cell-mediated immunity. The superior immunogenicity provided by live attenuated vaccines is due to their induction of a self-limiting and subclinical condition that simulates natural infection by virulent pathogens (usually with the same cell invasion and tissue tropism) and efficiently presents a full complement of immunogens [7]. Currently, only cattle brucellosis vaccines (RB51 or S19) are being used in most countries including the United States and most of Europe. However, safe and effective *Brucella *vaccines are desired for use in humans and many other animal species (e.g., goats, sheep, pigs, and wildlife). As a result, much research has been conducted and published in the area of *Brucella *vaccine research and development.

The mouse is the primary mammalian model used in the study of vaccine candidates including those against *Brucella *species [8]. Mice are genetically similar to humans with similar immune defenses and physiological mechanisms. The mouse genome sequence is available and has been well studied compared to other animal models. One critical issue to consider with vaccine animal studies is that many variables (e.g., vaccine doses, vaccination routes, and mouse strains and ages) can affect the outcomes of vaccine protection assays. A vaccine candidate may be concluded to be protective in a mouse study; however, using a different experimental protocol, the same vaccine candidate may be not protective anymore. Scientists often rely on previous research reports when formulating the experimental design for a future vaccine protection study. However, the large amount of literature reports often provides a variety of choices for setting the values of different variables. Sometimes conflicting results are found, causing confusion. It is frequently a challenge to design an experiment with optimal settings for different variables.

A vaccine protection investigation includes three processes: vaccination, pathogen challenge, and vaccine protection efficacy assessment [9]. A diverse assortment of variables may affect the results of vaccine protection. We hypothesize that some experimental variables play more important roles than others in determining the protection efficacy of any specific type of vaccines. In the Special Interest Group (SIG) meeting of "Bio-Ontologies 2010: Semantic Applications in Life Sciences" at the 18th annual conference on Intelligent Systems for Molecular Biology (ISMB 2010), we reported a preliminary study of an ontological representation of these variables using the Vaccine Ontology (VO) [10-12] and the Ontology for Biomedical Investigations (OBI) [13]. This ontological representation of statistical ANOVA analysis was used to analyze *Brucella *vaccine protections. In total, 151 mouse groups of *Brucella *vaccine protection investigations were collected from the literature and used to analyze 16 variables through ANOVA analysis. However, the focus of this study was on ontological representation of ANOVA statistical analysis instead of detailed meta-analysis of the vaccine protection studies. In addition, some of the relevant data in the domain of *Brucella *vaccine protection studies were not annotated. Whole-organism vaccines are generated based on whole organisms [14]. Two basic types of whole-organism vaccines exist. Live attenuated organisms are usually genetically generated through defined or undefined gene mutations of a wild type pathogen or by using close relatives to the target pathogen. Live vaccines cause a subclinical infection but still induce a protective immune response in the recipient. The other type of whole-organism vaccines is based on inactivation of whole intact organisms through high temperature (heating), chemical treatment, or physical treatment (*e.g*., gamma irradiation). The immunogenicity of nonliving whole-organism vaccines can be enhanced using vaccine adjuvants [15]. In this study, we focus on meta-analysis of mouse protection studies of whole-organism *Brucella *vaccines and vaccine candidates. To obtain the most valuable conclusions, all available literature data in this domain were annotated. A detailed statistical analysis was performed for each variable. Our meta-analysis provides results that facilitate practical experimental design in analysis of *Brucella *vaccine protection efficacy.

## Results

### Experimental design

Through review of the available literature, it was noted that the experimental design for testing vaccine efficacies varied for different pathogens and in different animal models. The mouse survival assay is one approach that measures the death of vaccinated or non-vaccinated mice after exposure to a virulent pathogen (e.g., virulent influenza, which can kill mice). In these studies, a vaccine's efficacy is judged by comparing the number of mouse deaths in the vaccinated and non-vaccinated groups [8]. However, some virulent pathogens do not kill mice but do colonize certain organs. In such cases, the colonization or survival of virulent pathogens in certain target organs (e.g., colon, spleen, liver, and lung) may be reduced in vaccinated mice. Accordingly, the colony or plaque forming units (CFU or PFU) reduction assay can be performed by using samples extracted from specific tissues. Virulent *Brucella *does not kill mice, instead surviving and replicating in the spleen and liver. Therefore, for *Brucella *vaccine studies, a CFU reduction assay in mice has been developed to measure vaccine effectiveness in mouse models. This *Brucella *CFU reduction assay also closely mirrors the normal disease course in humans and naturally infected animals. As an example, *Brucella abortus *cattle vaccine RB51 was used in a typical vaccine protection study as reported in reference [16]. In this study, BALB/c mice were vaccinated with live RB51 (1 × 10^8 ^CFU). Eight weeks later, vaccinated mice were challenged with virulent *B. abortus *strain 2308 (1 × 10^5 ^CFU). The CFU reduction in vaccinated mouse spleens was then counted and compared with those from non-vaccinated mice to determine vaccine protection [16].

**Figure 1:**
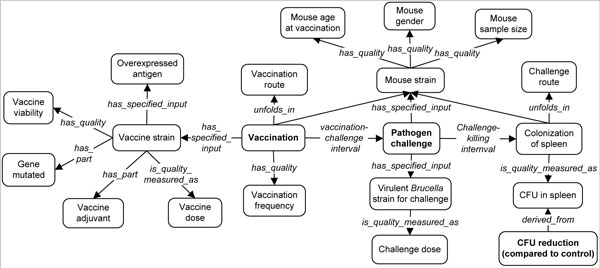
**Ontological representation of a vaccine protection assay and related variables**. The variables inside boxes are ontology terms primarily from the Vaccine Ontology (VO). The italicized relation terms are the terms from the Relation Ontology (RO) and the Ontology for Biomedical Investigations (OBI).

Similar studies were used to generate the data used in this meta-analysis. A list of variables was first preselected based on a systematic analysis of the vaccine protection studies. We have previously reported an ontological representation of many variables that exist during vaccine protection investigation [13]. From that preliminary study, we generated a simple but concise ontological representation showing how 20 variables were chosen based on the process pipeline of a typical *Brucella *vaccine protection investigation study (Figure 1). Four variables are associated with the host, *i.e*., mouse: Mouse strain, Mouse sex, Mouse age at the time of vaccination, and Mouse Sample Size. Five variables are related to the vaccine used for vaccination: Vaccine strain, Vaccine viability, Antigen overexpression in live *Brucella *strain carrier, Gene mutation, and Vaccine adjuvant. Three variables are related to the vaccination process: Vaccine dosage, Vaccination route, and Vaccination frequency. The variable Vaccination-challenge interval links the vaccination process with the challenge process. Three more variables, Challenge route, Challenge *Brucella *strain, Challenge dose, and Challenge-killing interval are associated with the challenge process. All these 17 variables are independent variables since their values can be manipulated or changed. With these independent variables established, three dependent variables can be measured and evaluated: the CFUs in spleen, CFU reduction, and the Protection significance level. The results of these dependent variables are the observed results of the independent variables being manipulated. It is noted that the significance of vaccine protection has two possible values: "protection" and "no protection". The assignment of the value depends on the statistical analysis obtained from the literature. Specifically, when a statistically significant result was achieved in a vaccine protection study group and was clearly described in a peer-reviewed paper, we assigned "protection" to this study group. Otherwise, "no protection" was assigned.

*Brucella*-related vaccinology is an active research area with more than 1300 peer-reviewed papers stored in PubMed. In this study, we chose to study only mouse protection data associated with whole-organism *Brucella *vaccines or vaccine candidates. This category includes live attenuated or inactivated vaccines or vaccine candidates. The inactivation can be generated by heat, chemicals, or gamma-irradiation. This decision avoids the issue of what to do with vaccine-related variables with no assignable value (*e.g*., Vaccine strain for a subunit vaccine). We have limited our focus to the mouse model for the following reasons: 1) most *Brucella *vaccine protection studies have been performed using the mouse model thereby resulting in a large body of available data, 2) while other animals models (*e.g*., rats, guinea pigs, and monkeys) have been used [17], the inclusion of the relatively small amount of data available for these models would increase the complexity of this meta-analysis, and 3) by focusing on a small specific research domain, we can spend a reasonable time to ensure an inclusion of all possible literature data. Over 100 publications investigated the vaccine protection efficacy of whole-organism *Brucella *vaccines in the mouse model. All these papers were annotated. Some papers were not used for various reasons, for example, lack of published values for one or more of the 20 variables. Eventually, 74 papers were included in our study, which contained complete and relevant experimental data for our meta-analysis. In total, data of 401 experimental mouse groups were manually annotated from these peer-reviewed publications. Each group of mice in a *Brucella *vaccine protection study reported in a peer-reviewed publication is considered as one experimental dataset.

With the experimental design and the collected data, a meta-analysis was performed. The raw data were first normalized by transformation. The normalized data was then analyzed by statistical methods including ANOVA. Our study indicates that many variables contribute to *Brucella *vaccine protection, while many do not.

### ANOVA statistics analysis identified variables contributing significantly to the final vaccine protection results

The data transformation procedure is described in the Methods section. The raw data and normalized data are provided in Additional file 1. The paper references used for the data collection are provided in Additional file 2. To analyze which variables contribute to the vaccine protection, the significance of vaccine protection is set as a dependent variable, and the other 18 variables are independent variables. An ANOVA analysis was performed and indicated that eight parameters significantly contribute to the vaccine protection (p-value < 0.05) (Table 1). These eight variables are: CFU reduction, Challenge route, Vaccine dosage, Vaccine strain, Vaccination route, Mouse strain, Vaccination frequency, and Challenge *Brucella *strain. The other 11 variables do not significantly contribute to the protection (p-value > 0.05).

**Table 1:** ANOVA results of 19 variables contributing to *Brucella *vaccine protection efficacy

#	Variables	Df	Sum Square	Mean Square	F value	P-value (>F)
**Variables with statistically significant contribution to protection (P-value < 0.05)**						
**1**	CFU Reduction	1	4.32	4.32	58.66	1.574E-13
**2**	Pathogen challenge route	1	2.20	2.20	29.90	8.278E-08
**3**	Vaccine dose	1	1.77	1.77	24.09	1.369E-06
**4**	CFU in spleen	1	0.92	0.92	12.49	4.591E-04
**5**	Challenge Pathogen Strain	1	0.85	0.85	11.54	0.0008
**6**	Mouse strain	1	0.58	0.58	7.86	0.0053
**7**	Vaccine strain	1	0.41	0.41	5.56	0.0188
**8**	Vaccination route	1	0.41	0.41	5.54	0.0190
**9**	Challenge-killing Interval	1	0.38	0.38	5.19	0.0232
**Variables without statistically significant contribution to protection (P-value > 0.05)**						
**10**	Vaccination Frequency	1	0.27	0.27	3.61	0.0581
**11**	Adjuvant	1	0.18	0.18	2.41	0.1215
**12**	Vaccination Challenge Interval	1	0.16	0.16	2.20	0.1385
**13**	Gene Mutation	1	0.12	0.12	1.68	0.1951
**14**	Mouse Age at Vaccination	1	0.07	0.07	0.97	0.3244
**15**	Sample Size	1	0.03	0.03	0.47	0.4928
**16**	Vaccine viability	1	0.02	0.02	0.34	0.5612
**17**	Antigen Overexpression	1	0.02	0.02	0.23	0.6299
**18**	Mouse Sex	1	0.01	0.01	0.10	0.7499
**19**	Challenge Dose	1	0.00	0.00	0.07	0.7988

**Note: **Nine variables were found to play a significant role (P-value < 0.05), and 10 variables were found not. **Df: **degree of freedom.

### Nine variables significantly contribute to *Brucella *vaccine protection

Each of the nine variables contributed significantly (P-value < 0.05) to *Brucella *vaccine protection. Each of these variables is examined here:

#### Variable 1: Vaccine strain

**Figure 2:**
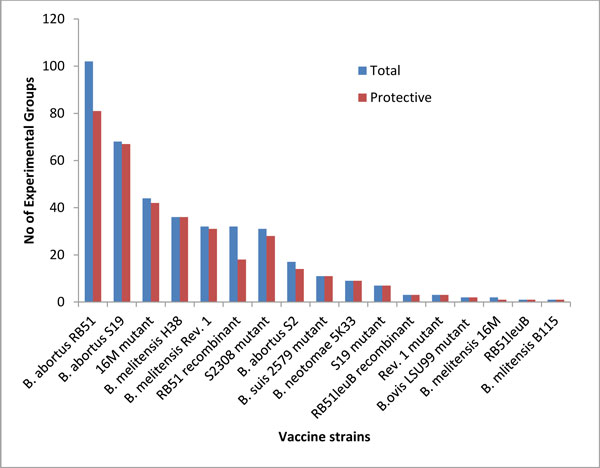
**The effect of vaccine strains on induction of protection in mice**. Total: all experimental groups are included; Protective: only those experimental groups showing protective results are included.

The variable "Vaccine strain" covers 17 different vaccine strains and one saline control (Figure 2). In every experimental study, saline (or PBS buffer) administration was always used as a negative control. These control groups never induced any protection. In our meta-analysis study, we did not include any saline injection experimental groups in our data analysis. However, the saline control has been used in every single experimental group study, since the CFUs in the control group were always used to calculate the values for the variable CFU reduction for each treatment group. Among the 17 vaccine strains, an immunization with nine strains, such as H38, RB51leuB, recombinant RB51leuB strains expressing protective *Brucella *antigens, *B. neotomae *strain 5K33, and *B. suis *2579 mutant (e.g., VTRS1), always generated significant protection in our record. The other eight vaccine strains did not induce significant protection in many studies. For example, *B. abortus *strains RB51 and S19 are two vaccine strains that have been used most frequently in various mouse vaccine protection studies. While both are effective vaccines, neither of them induced significant protection in all studies. Among all 165 RB51 experimental groups, 129 groups (78%) showed statistically significant protection. For the insignificance of RB51 in inducing protective immunity in mice, the use of different variable settings is the main reason:

a) ***Vaccination route***: Oral (PO) vaccination with RB51 is less effective than intraperitoneal (IP) vaccination [18]. The reduced vaccine efficacy is probably due to the lower persistence of RB51 *in vivo *after PO vaccination with RB51 [18]. It was found that intranasal (IN) vaccination with RB51 did not induce respiratory protection against intranasal pathogenic *Brucella *infection, even when a booster vaccination step was implemented [19]. However, another study found that oral administration of RB51 induced protection to mice orally infected with the virulent *B. abortus *strain 2308, but not to mice infected intraperitoneally [20].

b) ***Vaccine viability***: Killed RB51 does not seem to induce immunity in inoculated mice [21,22]. Even the addition of an IL-12 vaccine adjuvant does not influence protection induced by vaccination with killed RB51 [23,24].

c) ***Challenge route***: Mice vaccinated with RB51 and aerosol challenged with virulent *B. abortus *strain S2308 did not show a significant decrease in CFU in the spleen, liver, or the lungs compared to control mice [25].

d) ***Challenge pathogen strain***: When virulent *B. melitensis *strain 16M was used for mice challenge, RB51 was not effective in some of the time to protect RB51-vaccinated mice [26]. RB51 could not protect mice against virulent *B. suis *challenge. It is like that RB51 is a *B. abortus *vaccine only, and cannot provide sufficient cross protection against virulent *B. melitensis *or *B. suis *infections. However, a recombinant RB51*leuB *strain that overexpresses genes encoding Cu/Zn superoxide dismutase (SOD) and glycosyl-transferase (WboA) was effective in inducing protection in mice against infection with virulent *B. suis *[27].

Similar to RB51, IN administration of *B. abortus *vaccine strain 19 did not induce significant clearance of strain 2308 from spleen upon IN challenge infection compared to the control group [19]. Interestingly, this failure of S19 inducing significant protection in this case was the only report that demonstrated the insignificance of S19 in protection among all 77 experimental groups.

#### Variable 2: Mouse strain

**Figure 3:**
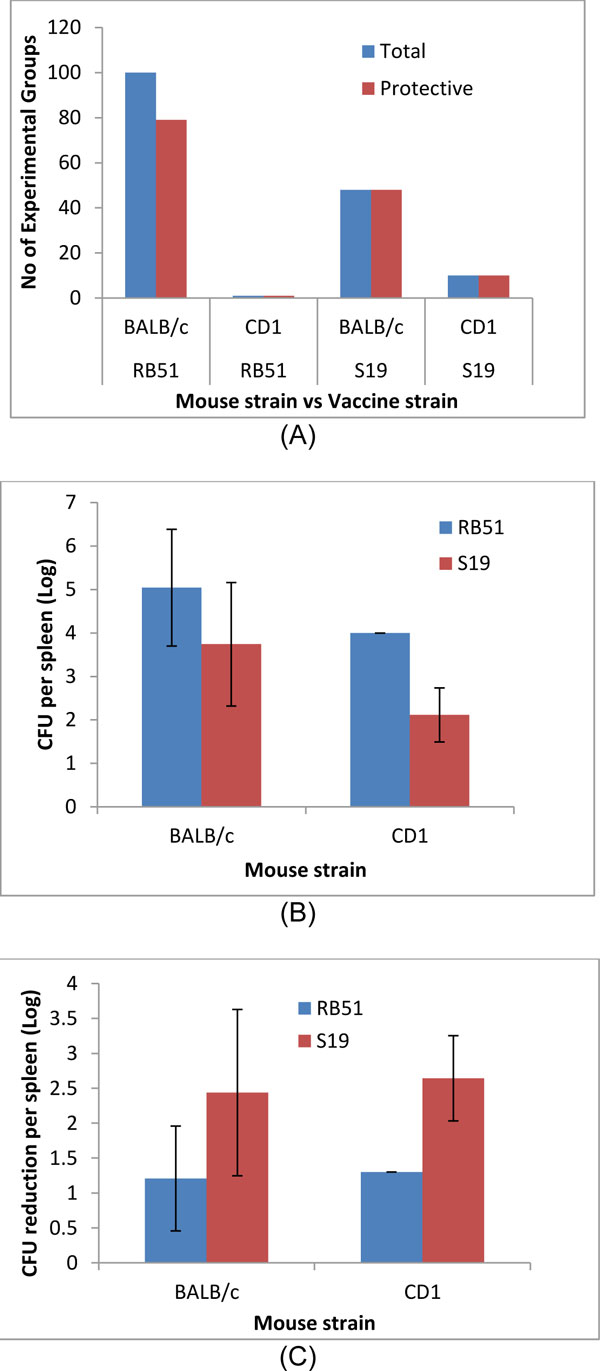
**The effects of vaccine strain and mouse strain on vaccine protection (A), CFUs (B), and CFU reduction (C) in mice**.

Seven different types of mouse strains have been used in *Brucella *vaccine protection studies: BALB/c, CD1, mixed/outbred, C57BL/6, OF1, 129/Sv, and Swiss Albino. The BALB/c mouse model is the most frequently used and has been used in 294 groups, with 254 groups showing protection in mice. CD1 is the secondly most frequently used mouse model, with a total of 70 groups used and 67 of them inducing protection. It is interesting that the BALB/c mice immunized with RB51 or S19 appear to reduced CFU and provide protection against challenge strain S2308 similar to CD1 mice immunized with RB51 or S19 (Figure 3A and 3B). One possible reason is that compared to outbred CD1 mice, inbred BALB/c mice are more sensitive to virulent S2308 infection. However, the general patterns of CFU reduction in RB51 or S19 in both mice types are similar. RB51-immunized mice induced approximately 1-1.5 log difference of CFU in the spleen in either mouse model. S19 induced 2-3 log CFU reduction in either mouse model (Figure 3C).

#### Variable 3 and 4: Vaccination route vs. pathogen challenge route

**Figure 4:**
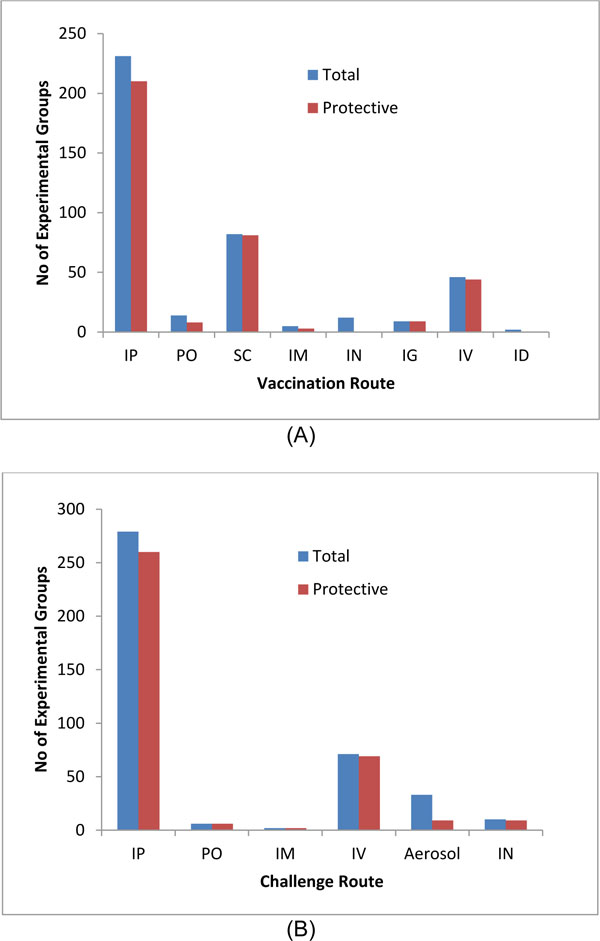
**The effects of vaccination route (A) and challenge route (B) on induction of protection in mice**.

Vaccination route and challenge route are two important variables for determining vaccine protection efficacy results. Among all vaccination routes used, the IP injection is the most common for *Brucella *vaccine protection studies (Figure 4A). Among all 223 experimental groups using IP vaccination, 205 groups induced significant protection. All studies with IM (intramuscular), IG (intragastric), and ID (intradermal) vaccination route induced protection. Most of SC (subcutaneous) and intravenous (IV) immunizations were successful in induction of protective immunity. No significant protection results in mouse spleen were found from the IN immunization route in all 12 experimental groups, indicating it is probably difficult to induce protection through IN immunization. For example, IN vaccination of mice with either RB51 or S19 was not able to induce significant clearance of virulent S2308 infection in spleen compared to saline control [19]. However, using IN vaccination, S19 (but not RB51) induced significant clearance of virulent S2308 in lung against IN challenge with virulent S2308 [19], indicating that S19 is more effective than RB51 in inducing respiratory protection. Oral (PO) vaccination appears not to be a favorable method in inducing protective immunity, with only 50% of chance of inducing protection. Therefore, the route of vaccination is an important variable to determine the protection efficacy even for those known *Brucella *vaccines (e.g., RB51).

Similarly, for virulent pathogen challenge, IP was by far the most used route. Among 271 groups using IP pathogen challenge protocol, 256 groups (94.4%) showed protection (Figure 4B). This rate is considered high. It appears extremely hard to induce protection against an aerosol challenge. Only 9 out of 33 groups (27.3%) using aerosol pathogen challenge induced significant protection. Among the 9 groups, all of them were achieved by IP vaccination with *B. abortus *or *B. melitensis *mutant strains, such as BAΔ*asp*24 or BMΔ*asp*24 unmarked mutant strain [28]. The other group that achieved protection against lung infection is through the IN vaccination with S19 as described in the above paragraph [19].

#### Variable 5: Vaccine dose

A large spread of values of vaccine dosage was observed, ranging from a CFU of 29 (1.46 log) to a CFU of 2 × 10^10 ^(10.3 log) (Additional file 1). The values of doses between different vaccines may differ dramatically. For example, a value of 0.5-5 × 10^8 ^CFUs of RB51 is typically used for RB51 protection study in mice [16,24]. However, the typical vaccination dose for S19 is 10^5 ^[29]. For any single vaccine, a larger dosage of vaccine induces better protective immunity. For example, mice are protected against IN challenge with virulent *B. melitensis *16M following oral vaccination with *B. melitensis *WR201 at a dose of 10^10 ^or 10^11 ^CFU. The protection level with a dose of 10^11 ^CFU is slightly better than that with a dosage of 10^10^. However, a dosage of 10^9 ^failed to provide a statistically significant protection [30].

#### Variable 6: Challenge strain

In total 17 *Brucella *strains have been used for the 401 groups of *Brucella *vaccine protection studies. Among them, *B. abortus *strain 2308 was used in 184 groups of mouse studies, with 153 studies generating significant protection. *B. abortus *strain S544 is the second most frequently used strain for mouse challenge. *B. melitensis *strain 16M was used in 52 groups.

#### Variable 7: Challenge-killing interval

This variable represents the time between a pathogen challenge and mouse killing for CFU measurement. The interval varies a lot in different studies, ranging from three days to 56 days. One to four weeks are most frequently used challenge-killing interval range. Among them, the most frequently used challenge-killing interval is the 14 day, which has been used in 199 experimental group studies. Thirty-nine of these studies did not generate protective results.

#### Variables 8 and 9: CFU vs. CFU reduction

The colony forming unit (CFU) of *Brucella *in spleen measures the number of live *Brucella *cells in a mouse spleen. Although lung and liver have also been tested, spleen is the most frequently used organ for CFU testing. The calculation of statistical significance of vaccine-induced protection in mice is typically performed by a *t*-test of comparing the CFUs of spleens between the treatment group and the control (saline or PBS) group.

The CFU reduction represents the relative reduction of CFU from treated group compared to the control group, statistically determines the protection level. Since the CFU counts may change depending on many variables such as the mouse type and the dosage of pathogen challenge, the CFU reduction is considered as a better indicator of the statistical significance of protection than CFU alone. Our analysis confirms this hypothesis. The CFU reduction has much more significant P-value than the CFU variable (Table 1).

### Ten variables do not significantly contribute to *Brucella *vaccine protection

Ten variables do not significantly contribute to *Brucella *vaccine protection (P-value > 0.05). Each of them is examined below in detail.

#### (1) Antigen overexpression

Very few vaccines recorded in our study included an overexpressed *Brucella *antigen. *B. abortus *vaccine RB51 is able to overexpress homologous *Brucella *proteins as a way to enhance its vaccine efficacy [31,32]. RB51 has been used to overexpress Cu/Zn superoxide dismutase (SOD) [31] and express *Brucella *glycosyl-transferase (*WboA*) [33] to achieved enhanced vaccine efficacy. RB51*leuB *is an unmarked *leuB *mutant of RB51 [34]. The *leuB *gene, encoding isopropyl malate dehydrogenase, is essential for the biosynthesis of leucine in *B. abortus*. The resultant *leuB *auxotroph cannot grow in leucine-deficient conditions. Complementation of the *leuB *auxotroph with a plasmid carrying the wild-type *leuB *gene allows its survival in leucine-deficient minimal medium and under nutrient-limiting conditions *in vivo*. The selective growth pressure provides a mechanism for the maintenance of the plasmid. The environmentally safe leucine auxotroph of strain RB51 was used to over-express genes encoding homologous proteins: L7/L12 ribosomal protein, Cu/Zn superoxide dismutase (SOD) and glycosyl-transferase (WboA). Mice vaccinated with RB51leu*B*/SOD/WboA were significantly better protected than those that were vaccinated with either strain RB51leuB/SOD or RB51leu*B*/SOD/L7/L12 [27]. It was also recently found that non-pathogenic *B. neotomae *strain 5K33 can be used to overexpress homologous *Brucella *proteins [35]. Recombinant *B. neotomae *strains overexpressing SOD and/or BP26 were found to induce protection against challenge with virulent *B. abortus *2308, *B. melitensis *16 M, and *B. suis *1330 [35]. However, the approach of overexpression of protective antigen does not guarantee enhanced protection. For example, RB51*leuB*/SOD or RB51*leuB*/SOD/L7/L12 does not provide statistically better protection than RB51*leuB *when BALB/c mice were challenged with virulent strain *B. suis *1330 [27]. The addition of protective antigen would seem to induce synergy and enhanced protection; however, one antigen may hinder the amount of the other preventing sufficient presentation to the immune system.

#### (2) Gene mutation

A virulence factor can be mutated in virulent pathogen, leading to the possible generation of a live attenuated vaccine. Eighteen *Brucella *virulence factors have been reported to be used for generation of live attenuated vaccines. These 18 virulence factors include: *asp24, bp26, exsA, manA, manB*, *mucR*, *omp25, omp31, p39, pgk, pgm, purE, purK, virB2, vjbR, wbkA*, *wboA*, and *znuA*. Among these genes, *manA *and *manB *were mutated together in *B. abortus *strain 2308 or *B. melitensis *strain 16M [36]. Two other genes *purE *and *purK *were both mutated in *B. melitensis *strain 16M [30]. The other 14 genes were mutated individually in different parent *Brucella *strains. Most of these mutants induced protection against virulent *Brucella *challenge under different experimental conditions of the mouse model. However, while RB51*WboA *provided protection in 11 case studies, RB51WboA failed to induce protection against intranasal challenge with virulent *B. abortus challenge *[19]. Therefore, a vaccine that works out in one experimental condition may not work in another experimental condition.

#### (3) Vaccine adjuvant

Live attenuated vaccines usually do not need any vaccine adjuvant to boost the induction of antigen-specific immune response. In total, 357 groups of vaccine experiments did not use any vaccine adjuvant. Among them, 317 induced protection in the mouse model. However, inactivated whole-organism vaccines may need to use vaccine adjuvant to facilitate the induction of adaptive immunity. IFA and oil adjuvants have been used in 5 and 12 groups of vaccine protection studies, respectively and all of them induced protection. Among the six groups of vaccine protection studies that used IL-12 as vaccine adjuvants, only 3 (50%) of them demonstrated protection [23,24]. IL-12 is an important cytokine for Th1 cell development and proliferation and has exhibited an enhanced protective effect on *Listeria *and *Leishmania *infections but it appears that in the context of *Brucella*, exogenous IL-12 is indispensable. Statistically, the addition of an adjuvant to a vaccine does not affect the efficacy of a vaccine (Table 1). However, since almost all vaccines that were examined did not include an adjuvant, the data might well be skewed.

#### (4) Vaccine viability

Whole-organism *Brucella *vaccines and vaccine candidates are live attenuated, irradiated, or killed by heat or chemicals. Live virulent *Brucella *strains cannot be used as vaccines since they are not safe. Irradiated and killed vaccines are both classified as inactivated vaccine type. In our annotated data record, there exist 357 experimental groups that use live attenuated vaccines, making up nearly the entire amount of vaccines. Among them, 318 induced protection against virulent *Brucella *infection in corresponding vaccine protection studies. Eleven out of 16 irradiated vaccines provided protection in the mouse model. Only two out of 36 killed *Brucella *vaccine candidates failed to generate protection. This does not mean that killed vaccines are more effective than live attenuated vaccines. Instead, the data suggest that killed *Brucella *vaccine candidates can be protective giving the appropriate vaccine formula (e.g., having adjuvants) and experimental conditions.

#### (5) Mouse sex

The female sex was virtually the only sex used in the studies. There has not been a single group of vaccine protection that specifically claimed to use male mice. Only two mouse groups used both female and male mice [37]. Therefore, the data is not sufficient to support that mouse sex does not change the results.

#### (6) Mouse age at vaccination

Similar percent significances between the <69 day and the 69-224 day-old mice were identified. Specifically, 90.6% of groups (289 out of 319) of vaccine protection studies used mice under < 69 days old. In comparison, 80% of groups (48 out of 60) used mice with 69-226 days old. The difference between these two groups was not significantly different.

#### (7) Mouse sample size per experimental group

In our record, 3-134 mice per experimental group were used in various studies. The most frequently used sample size is 5 mice per group, accounting for 214 experimental group studies. Among these groups, 26 groups did not result in any protective response (P-value > 0.05).

#### (8) Vaccination frequency

In our records, a *Brucella *vaccine or vaccine candidate was used in vaccination for once, twice, or three times. This means that the variable "Vaccine Frequency" has three values: 1, 2, or 3. It appears intuitive that the greater the number of injections of the vaccine, the more effective the vaccine is. However, based on the present analysis, one vaccination is the most efficient frequency of vaccination. This is because one vaccination with a live attenuated *Brucella *vaccine (e.g., RB51 or S19) is sufficient to induce significant protection in mouse models. Killed *Brucella *vaccine candidates tend not to induce protection in mice. The usage of two or three vaccinations is often required for those inactivated vaccine candidates or those live attenuated vaccines that were administrated differently (*e.g*., PO administration instead of traditional IP injection). The frequency of 1 occurred for 351 groups, 90% of which showed significant protection. When a vaccine was used twice, 79.2% of cases showed significant protection. Lastly, when a vaccine was used three times, 100% of protection was obtained.

#### (9) Vaccination-challenge interval

Different vaccination-challenge intervals have been applied, ranging from 7 days to 210 days. The top three vaccination-challenge intervals are 30 days (44 cases), 42 days (53 cases), and 56 days (64 cases). Among these intervals, 75% to 93% of vaccine protection cases provided significant protection. Our results showed that the vaccination-challenge interval is not statistically significant factor for the vaccine protection efficacy outcome. This result allows us to comfortably rearrange our experimental design in terms of vaccination-challenge intervals.

#### (10) Challenge dose

The challenge doses used in most vaccine protection studies ranged from 10^4 ^to 10^6^. Specifically, 247, 75, and 25 vaccine studies used challenge doses with the scale of 10^4^, 10^5^, and 10^6^, respectively. Correspondingly, 212, 71, and 22 groups induced protection, respectively. It appears that the results of protections did not change much in these groups. A higher amount of virulent challenge dose turned to increase the amount of *Brucella *remaining in the spleen (i.e., CFU in the spleen). However, a higher CFU does not equal to a higher CFU reduction. The reduction of CFUs in spleen is a factor better correlated with protection efficacy than the absolution number of CFUs in spleen.

## Discussion

Large amounts of literature data have been accumulated in almost every biomedical domain. It is difficult to have a comprehensive understanding of a research domain without taking into account all possible data available in the literature. Our study proves that meta-analysis is a valid approach to study biomedical problems. New knowledge and novel hypotheses may be generated from the study of literature reports. It is frequent that we could not find out the exact values for many variables from many literature reports. This prevents us from including many papers in our study. Therefore, it is critical for the authors of literature papers to describe experimental design and protocols in details.

Even for known *Brucella *vaccines (*e.g*., RB51), proper experimental design for a vaccine protection study is important to generate protection (except for testing different experimental conditions). Inhalation of aerosolized *Brucella *is one of the major routes of disease transmission in humans [16]. Therefore, to successfully control brucellosis, it is crucial to identify those experimental factors that may influence vaccine protection.

There are some variables which are not critical and some variations are fine and do not affect the final protection outcome. However, to claim they are insignificant is relative to the data collected from this study. For many variables (e.g., Mouse Sex), the data presented are biased and often insufficient to draw a conclusion. To overcome this obstacle, we can include more datasets that cover these other factors and try to have similar or balanced numbers for different factors.

This study also suggests new foci for further research into the protective mechanisms against *Brucella*. Many hypotheses can be generated from the meta-analysis. For example, most of the studies using an aerosol challenge used the IN vaccination route. However, IN vaccination route may not be the most effective way to induce protection against aerosol pathogen challenge. This is indicated by the low rate of success from previous reports. However, it has been found many times that systematic immunization, including IP and IN immunization, may induce strong protection against respiratory infection of virulent *Brucella*. More evidences can be found from non-whole-organism *Brucella *vaccine studies. For example, IN immunization of mice with a *B. melitensis *lipopolysaccharide subunit vaccine provided significant protection against disseminated infection of the spleen and liver but SC immunization of mice with the vaccine conferred significant protection against infection of the spleen, liver, and lungs [38]. The reasons of this phenomenon may be complex. The mucus acts as a "physical barrier" as a means for preventing foreign materials to enter the host body. One possible explanation is that *Brucella *vaccines may not effectively penetrate into mucosal physical barriers (*e.g*., nose). Therefore, *Brucella *antigens cannot efficiently be presented to the antigen presenting cells, including dendritic cells and macrophages [39].

Meta-analysis is data analysis method applied to summarizing research findings of individual studies at both quantitative and qualitative levels [40]. Meta-analysis combines several studies and thus is less influenced by local findings from single studies. Meta-analysis can be used to show whether the results are more varied than what is expected from the sample diversity. Statistical testing of overall factors and effect size parameters in related studies can be performed using meta-analysis. Meta-analysis is able to control for between-study variation; Moderators can be included in meta-analysis to explain variation. Meta-analysis has higher statistical power to detect an effect than single studies with "n = 1 sized study sample". Meta-analysis can also be used to address information overload since the high number of articles has been published each year. Besides ANOVA, there are many different statistical methods for meta-analysis [41-43]. ANOVA is used in this study because it is relatively simple and fits in with our research objectives.

The whole process of meta-analysis design and data annotation is very time-consuming. It would be beneficial to generate a general systematic meta-analysis design pattern and develop advanced tools to implement biomedical meta-analysis more effectively. Recently we have initiated the development of community-driven Vaccine Ontology (VO; http://www.violinet.org/vaccineontology) [10-12]. VO is a controlled vocabulary of terms and relations that pertains to the vaccine domain. While seemingly straightforward, the standardization of vaccine-associated terms and the relations between these terms requires multi-field knowledge and expertise on many different levels. The Vaccine Ontology (VO), combined with other sister ontologies (*e.g*., the Ontology for Biomedical Investigations or OBI), can be used to represent the diverse variables used in analysis of animal responses to vaccination (Figure 1). Not only these representations can be understood by humans, but they can also be parseable and understood by computers. Therefore, new tools can be developed to understand and analyze the collected literature data, thereby allowing "computer-assisted automated reasoning". For example, advanced ontology-based literature mining tools may be developed to automate many processes of retrieving and analyzing data from the literature. Novel hypotheses may also be generated to provide new avenues for future research. These activities will increase the effectiveness of meta-analysis of different variables to uncover those important to vaccine-induced protection studies or other biomedical domains [44].

Our approach represents a genetic literature-based meta-analysis of biological experiments in the vaccine protection investigation. Through meta-analysis we were able to identify variables that significantly contributed to *Brucella *vaccine protection. The method reported in this study can be applied to analyze variables critical for vaccine protection against other infectious diseases (*e.g*., AIDS, malaria, and tuberculosis). Approximately 3,000 vaccines and the data of protection studies involving them have been manually curated and stored in the Vaccine Investigation and Online Information Network (VIOLIN) vaccine database system [45]. This approach can also be applied to study other pathogen-vaccine interactions in specific animal models, as outlined in VIOLIN.

## Conclusions

A known vaccine may not induce protection if experimental conditions are not optimized. Our studies identified several variables that play critical roles in inducing protective immunity in the mouse model, and many other variables do not. New knowledge and hypotheses were obtained. For example, *B. abortus *cattle vaccine RB51 may not induce protection in mice if the values of many experimental variables (e.g., vaccination route, vaccine viability, and challenge pathogen strain) change. Our study found that IN or PO immunization tends to induce low protection, while IP or SC injection may induce systemic immune response and protect against aerosol or IN pathogen challenge. The meta-analysis provides a unique systematic approach towards better understanding of vaccine-induced protection to brucellosis.

## Methods

### Curation process of vaccine animal response data

As of December 12, 2011, 124 papers were identified through a PubMed search for "*Brucella *vaccine mice challenge". These peer-reviewed research papers were manually curated to identify variables and extract values taken by these variables potentially important for vaccine protection efficacy investigation.

### Data transformation

The following six variables kept the continuous values: vaccine dose, challenge pathogen dose, vaccination-challenge interval, challenge-killing interval, CFUs, CFU reduction. Among them, vaccination-challenge interval uses the unit of day, and log10 transformation was applied for the other four variables. Other than these five variables, the raw data of 14 other variables in the *Brucella *vaccine protection meta-analysis was transformed to discretized data using a data discretization process. For example, the variable "Mouse Sex" has two values: female and male. During the data discretization step, the number string values, female and male, were discretized to two discrete digital values 0 and 1, respectively.

### General data analysis

Microsoft Excel program was used for generating 2D plot figures. Microsoft Excel was also used to calculate the sums, averages, and standard deviations for this meta-analysis.

### ANOVA statistical analysis of *Brucella *vaccine protection results

The analysis of variance (ANOVA) was first used to analyze which variables contributed significantly to the *Brucella *protection efficacy. R program was used to implement ANOVA analysis of the transformed data described above. Specifically, ANOVA for linear model fits was used. The predictive model is "Protection-level ~." indicating we are testing how each other variable affects the protection level. This linear model representation can be understood and processed by statistical software pro-grams such as R programming.

## List of abbreviations used

ANOVA: analysis of variance; ID: Intradermal route; IN: Intranasal route; IP: Intraperitoneal route; IV: Intravenous route; PO: Per oral (or *Per os*) route; SC: Subcutaneous route; VIOLIN: the Violin Investigation and OnLine Information Network; VO: Vaccine Ontology.

## Competing interests

The authors declare that they have no competing interests.

## Authors' contributions

TT: Project design, literature data annotation, data analysis, and manuscript drafting. OT: Literature data annotation and data analysis. YL, SS, and DNB: Literature data annotation. ZX: Statistics data analyses. YH: Project design and management, data interpretation, and manuscript drafting.

## Supplementary Material

Additional file 1 **The curated data used for meta-analysis**. Click here for file

Additional file 2 **The references of peer-reviewed journal papers that were used for collecting data in the meta-analysis study**. Click here for file

## Declarations

This article has been published as part of *BMC Bioinformatics *Volume 14 Supplement 6, 2013: Selected articles from the 10th International Conference on Artificial Immune Systems (ICARIS). The full contents of the supplement are available online at http://www.biomedcentral.com/bmcbioinformatics/supplements/14/S6.
