# Supplementary material for: Meta-analysis of variables affecting mouse protection efficacy of whole organism Brucella vaccines and vaccine candidates
Source: BMC Bioinformatics. 2013 Apr 17;14(Suppl 6):S3. doi: 10.1186/1471-2105-14-S6-S3 (PMC3633026; doi:10.1186/1471-2105-14-S6-S3)
Supplement: Additional file 2 — The references of peer-reviewed journal papers that were used for collecting data in the meta-analysis study. [file 1471-2105-14-S6-S3-S2.doc]

**Here is the list of 76 references** **that are used in this meta-analysis:**

1.	Briones G, Inon de Iannino N, Roset M, Vigliocco A, Paulo PS, Ugalde RA: ***Brucella* *abortus* cyclic beta-1,2-glucan mutants have reduced virulence in mice and are defective in intracellular replication in HeLa cells**. *Infect Immun* 2001, **69**(7):4528-4535.

2.	Adone R, Ciuchini F, Marianelli C, Tarantino M, Pistoia C, Marcon G, Petrucci P, Francia M, Riccardi G, Pasquali P: **Protective properties of rifampin-resistant rough mutants of *Brucella* *melitensis***. *Infect Immun* 2005, **73**(7):4198-4204.

3.	Adone R, Francia M, Ciuchini F: **Evaluation of *Brucella* *melitensis* B115 as rough-phenotype vaccine against *B. melitensis* and *B.* ovis infections**. *Vaccine* 2008, **26**(38):4913-4917.

4.	Al-Mariri A: **Protection of BALB/c mice against *Brucella* *melitensis* 16 M infection induced by vaccination with live Escherchia coli expression *Brucella* P39 protein**. *Vaccine* 2010, **28**(7):1766-1770.

5.	Al-Mariri A, Tibor A, Mertens P, De Bolle X, Michel P, Godfroid J, Walravens K, Letesson JJ: **Induction of immune response in BALB/c mice with a DNA vaccine encoding bacterioferritin or P39 of *Brucella* spp**. *Infect Immun* 2001, **69**(10):6264-6270.

6.	Arenas-Gamboa AM, Ficht TA, Kahl-McDonagh MM, Gomez G, Rice-Ficht AC: **The *Brucella* *abortus* S19 DeltavjbR live vaccine candidate is safer than S19 and confers protection against wild-type challenge in BALB/c mice when delivered in a sustained-release vehicle**. *Infect Immun* 2009, **77**(2):877-884.

7.	Arenas-Gamboa AM, Rice-Ficht AC, Kahl-McDonagh MM, Ficht TA: **Protective efficacy and safety of *Brucella* *melitensis* 16MDeltamucR against intraperitoneal and aerosol challenge in BALB/c mice**. *Infect Immun* 2011, **79**(9):3653-3658.

8.	Boschiroli ML, Cravero SL, Arese AI, Campos E, Rossetti OL: **Protection against infection in mice vaccinated with a *Brucella* *abortus* mutant**. *Infect Immun* 1997, **65**(2):798-800.

9.	Bosseray N: **Immunity to *Brucella* in mice vaccinated with a fraction (F8) or a killed vaccine (H38) with or without adjuvant. Level and duration of immunity in relation to dose of vaccine, recall injection and age of mice**. *Br J Exp Pathol* 1978, **59**(4):354-365.

10.	Bosseray N: **Colonization of mouse placentas by *Brucella* *abortus* inoculated during pregnancy**. *Br J Exp Pathol* 1980, **61**(4):361-368.

11.	Bosseray N: **Vaccine and serum-mediated protection against *Brucella* infection of mouse placenta**. *Br J Exp Pathol* 1983, **64**(6):617-625.

12.	Bosseray N, Plommet M: ***Brucella* *suis* S2, *Brucella* *melitensis* Rev. 1 and *Brucella* *abortus* S19 living vaccines: residual virulence and immunity induced against three *Brucella* species challenge strains in mice**. *Vaccine* 1990, **8**(5):462-468.

13.	Cabrera A, Saez D, Cespedes S, Andrews E, Onate A: **Vaccination with recombinant Semliki Forest virus particles expressing translation initiation factor 3 of *Brucella* *abortus* induces protective immunity in BALB/c mice**. *Immunobiology* 2009, **214**(6):467-474.

14.	Campos E, Cravero SL, Delgui L, Mora I, Kahn N, Arese AI, Rossetti OL: ***Brucella* *abortus* INTA2, a novel strain 19 (Delta)bp26::luc (Delta)bmp18 double mutant lacking drug resistance markers**. *Vet Microbiol* 2002, **87**(1):1-13.

15.	Cassataro J, Velikovsky CA, Bruno L, Estein SM, de la Barrera S, Bowden R, Fossati CA, Giambartolomei GH: **Improved immunogenicity of a vaccination regimen combining a DNA vaccine encoding *Brucella* *melitensis* outer membrane protein 31 (Omp31) and recombinant Omp31 boosting**. *Clin Vaccine Immunol* 2007, **14**(7):869-874.

16.	Cespedes S, Andrews E, Folch H, Onate A: **Identification and partial characterisation of a new protective antigen of *Brucella* *abortus***. *J Med Microbiol* 2000, **49**(2):165-170.

17.	Clapp B, Skyberg JA, Yang X, Thornburg T, Walters N, Pascual DW: **Protective live oral brucellosis vaccines stimulate Th1 and th17 cell responses**. *Infect Immun* 2011, **79**(10):4165-4174.

18.	Cloeckaert A, Jacques I, Grillo MJ, Marin CM, Grayon M, Blasco JM, Verger JM: **Development and evaluation as vaccines in mice of *Brucella* *melitensis* Rev.1 single and double deletion mutants of the bp26 and omp31 genes coding for antigens of diagnostic significance in ovine brucellosis**. *Vaccine* 2004, **22**(21-22):2827-2835.

19.	Commander NJ, Spencer SA, Wren BW, MacMillan AP: **The identification of two protective DNA vaccines from a panel of five plasmid constructs encoding *Brucella* *melitensis* 16M genes**. *Vaccine* 2007, **25**(1):43-54.

20.	Corbel MJ: **The immunogenic activity of ribosomal fractions derived from *Brucella* *abortus***. *J Hyg (Lond)* 1976, **76**(1):65-74.

21.	Delpino MV, Estein SM, Fossati CA, Baldi PC, Cassataro J: **Vaccination with *Brucella* recombinant DnaK and SurA proteins induces protection against *Brucella* *abortus* infection in BALB/c mice**. *Vaccine* 2007, **25**(37-38):6721-6729.

22.	Edmonds MD, Cloeckaert A, Elzer PH: ***Brucella* species lacking the major outer membrane protein Omp25 are attenuated in mice and protect against *Brucella* *melitensis* and *Brucella* ovis**. *Vet Microbiol* 2002, **88**(3):205-221.

23.	Estevan M, Gamazo C, Grillo MJ, Del Barrio GG, Blasco JM, Irache JM: **Experiments on a sub-unit vaccine encapsulated in microparticles and its efficacy against *Brucella* *melitensis* in mice**. *Vaccine* 2006, **24**(19):4179-4187.

24.	Gonzalez-Smith A, Vemulapalli R, Andrews E, Onate A: **Evaluation of *Brucella* *abortus* DNA vaccine by expression of Cu-Zn superoxide dismutase antigen fused to IL-2**. *Immunobiology* 2006, **211**(1-2):65-74.

25.	Grillo MJ, Manterola L, de Miguel MJ, Munoz PM, Blasco JM, Moriyon I, Lopez-Goni I: **Increases of efficacy as vaccine against *Brucella* *abortus* infection in mice by simultaneous inoculation with avirulent smooth bvrS/bvrR and rough wbkA mutants**. *Vaccine* 2006, **24**(15):2910-2916.

26.	Hamdy ME, El-Gibaly SM, Montasser AM: **Comparison between immune responses and resistance induced in BALB/c mice vaccinated with RB51 and Rev. 1 vaccines and challenged with *Brucella* *melitensis* bv. 3**. *Vet Microbiol* 2002, **88**(1):85-94.

27.	High KP, Prasad R, Marion CR, Schurig GG, Boyle SM, Sriranganathan N: **Outcome and immune responses after *Brucella* *abortus* infection in young adult and aged mice**. *Biogerontology* 2007, **8**(5):583-593.

28.	Izadjoo MJ, Bhattacharjee AK, Paranavitana CM, Hadfield TL, Hoover DL: **Oral vaccination with *Brucella* *melitensis* WR201 protects mice against intranasal challenge with virulent *Brucella* *melitensis* 16M**. *Infect Immun* 2004, **72**(7):4031-4039.

29.	Jimenez de Bagues MP, Elzer PH, Jones SM, Blasco JM, Enright FM, Schurig GG, Winter AJ: **Vaccination with *Brucella* *abortus* rough mutant RB51 protects BALB/c mice against virulent strains of *Brucella* *abortus*, *Brucella* *melitensis*, and *Brucella* ovis**. *Infect Immun* 1994, **62**(11):4990-4996.

30.	Jimenez de Bagues MP, Marin CM, Barberan M, Blasco JM: **Evaluation of vaccines and of antigen therapy in a mouse model for *Brucella* ovis**. *Vaccine* 1993, **11**(1):61-66.

31.	Kahl-McDonagh MM, Arenas-Gamboa AM, Ficht TA: **Aerosol infection of BALB/c mice with *Brucella* *melitensis* and *Brucella* *abortus* and protective efficacy against aerosol challenge**. *Infect Immun* 2007, **75**(10):4923-4932.

32.	Kahl-McDonagh MM, Ficht TA: **Evaluation of protection afforded by *Brucella* *abortus* and *Brucella* *melitensis* unmarked deletion mutants exhibiting different rates of clearance in BALB/c mice**. *Infect Immun* 2006, **74**(7):4048-4057.

33.	Kaushik P, Singh DK, Kumar SV, Tiwari AK, Shukla G, Dayal S, Chaudhuri P: **Protection of mice against *Brucella* *abortus* 544 challenge by vaccination with recombinant OMP28 adjuvanted with CpG oligonucleotides**. *Vet Res Commun* 2010, **34**(2):119-132.

34.	Lee IK, Olsen SC, Bolin CA: **Effects of exogenous recombinant interleukin-12 on immune responses and protection against *Brucella* *abortus* in a murine model**. *Can J Vet Res* 2001, **65**(4):223-228.

35.	Lee IK, Olsen SC, Kehrli M, Bolin CA: **The adjuvant effect of a single dose of interleukin-12 on murine immune responses to live or killed *Brucella* *abortus* strain RB51**. *Can J Vet Res* 1999, **63**(4):284-287.

36.	Magnani DM, Harms JS, Durward MA, Splitter GA: **Nondividing but metabolically active gamma-irradiated *Brucella* *melitensis* is protective against virulent *B. melitensis* challenge in mice**. *Infect Immun* 2009, **77**(11):5181-5189.

37.	Mahajan NK, Kulshreshtha RC, Malik G, Dahiya JP: **Immunogenicity of major cell surface protein(s) of *Brucella* *melitensis* Rev 1**. *Vet Res Commun* 2005, **29**(3):189-199.

38.	Monreal D, Grillo MJ, Gonzalez D, Marin CM, De Miguel MJ, Lopez-Goni I, Blasco JM, Cloeckaert A, Moriyon I: **Characterization of *Brucella* *abortus* O-polysaccharide and core lipopolysaccharide mutants and demonstration that a complete core is required for rough vaccines to be efficient against *Brucella* *abortus* and *Brucella* ovis in the mouse model**. *Infect Immun* 2003, **71**(6):3261-3271.

39.	Montaraz JA, Winter AJ: **Comparison of living and nonliving vaccines for *Brucella* *abortus* in BALB/c mice**. *Infect Immun* 1986, **53**(2):245-251.

40.	Moustafa D, Garg VK, Jain N, Sriranganathan N, Vemulapalli R: **Immunization of mice with gamma-irradiated *Brucella* neotomae and its recombinant strains induces protection against virulent *B. abortus*, *B. melitensis*, and *B. suis* challenge**. *Vaccine* 2011, **29**(4):784-794.

41.	Olsen SC, Waters WR, Stoffregen WS: **An aerosolized *Brucella* spp. challenge model for laboratory animals**. *Zoonoses Public Health* 2007, **54**(8):281-285.

42.	Onate AA, Cespedes S, Cabrera A, Rivers R, Gonzalez A, Munoz C, Folch H, Andrews E: **A DNA vaccine encoding Cu,Zn superoxide dismutase of *Brucella* *abortus* induces protective immunity in BALB/c mice**. *Infect Immun* 2003, **71**(9):4857-4861.

43.	Onate AA, Donoso G, Moraga-Cid G, Folch H, Cespedes S, Andrews E: **An RNA vaccine based on recombinant Semliki Forest virus particles expressing the Cu,Zn superoxide dismutase protein of *Brucella* *abortus* induces protective immunity in BALB/c mice**. *Infect Immun* 2005, **73**(6):3294-3300.

44.	Onate AA, Vemulapalli R, Andrews E, Schurig GG, Boyle S, Folch H: **Vaccination with live Escherichia coli expressing *Brucella* *abortus* Cu/Zn superoxide dismutase protects mice against virulent *B. abortus***. *Infect Immun* 1999, **67**(2):986-988.

45.	Pakzad I, Rezaee A, Rasaee MJ, Hossieni AZ, Tabbaraee B, Kazemnejad A: **Protection of BALB/C mice against *Brucella* *abortus* 544 challenge by vaccination with combination of recombinant human serum albumin-l7/l12 (*Brucella* *abortus* ribosomal protein) and lipopolysaccharide**. *Roum Arch Microbiol Immunol* 2010, **69**(1):5-12.

46.	Pasquali P, Adone R, Gasbarre LC, Pistoia C, Ciuchini F: **Mouse cytokine profiles associated with *Brucella* *abortus* RB51 vaccination or *B. abortus* 2308 infection**. *Infect Immun* 2001, **69**(10):6541-6544.

47.	Pasquali P, Rosanna A, Pistoia C, Petrucci P, Ciuchini F: ***Brucella* *abortus* RB51 induces protection in mice orally infected with the virulent strain *B. abortus* 2308**. *Infect Immun* 2003, **71**(5):2326-2330.

48.	Pasquevich KA, Estein SM, Garcia Samartino C, Zwerdling A, Coria LM, Barrionuevo P, Fossati CA, Giambartolomei GH, Cassataro J: **Immunization with recombinant *Brucella* species outer membrane protein Omp16 or Omp19 in adjuvant induces specific CD4+ and CD8+ T cells as well as systemic and oral protection against *Brucella* *abortus* infection**. *Infect Immun* 2009, **77**(1):436-445.

49.	Pasquevich KA, Ibanez AE, Coria LM, Garcia Samartino C, Estein SM, Zwerdling A, Barrionuevo P, Oliveira FS, Seither C, Warzecha H *et al*: **An oral vaccine based on U-Omp19 induces protection against *B. abortus* mucosal challenge by inducing an adaptive IL-17 immune response in mice**. *PLoS One* 2011, **6**(1):e16203.

50.	Plommet M, Plommet AM: **Immunity to *Brucella* *abortus* induced in mice by popliteal lymph node restricted strain 19 vaccination**. *Ann Rech Vet* 1989, **20**(1):73-81.

51.	Rajasekaran P, Seleem MN, Contreras A, Purwantini E, Schurig GG, Sriranganathan N, Boyle SM: ***Brucella* *abortus* strain RB51 leucine auxotroph as an environmentally safe vaccine for plasmid maintenance and antigen overexpression**. *Appl Environ Microbiol* 2008, **74**(22):7051-7055.

52.	Rajasekaran P, Surendran N, Seleem MN, Sriranganathan N, Schurig GG, Boyle SM: **Over-expression of homologous antigens in a leucine auxotroph of *Brucella* *abortus* strain RB51 protects mice against a virulent *B. suis* challenge**. *Vaccine* 2011, **29**(17):3106-3110.

53.	Rosinha GM, Freitas DA, Miyoshi A, Azevedo V, Campos E, Cravero SL, Rossetti O, Splitter G, Oliveira SC: **Identification and characterization of a *Brucella* *abortus* ATP-binding cassette transporter homolog to Rhizobium meliloti ExsA and its role in virulence and protection in mice**. *Infect Immun* 2002, **70**(9):5036-5044.

54.	Rosinha GM, Myioshi A, Azevedo V, Splitter GA, Oliveira SC: **Molecular and immunological characterisation of recombinant *Brucella* *abortus* glyceraldehyde-3-phosphate-dehydrogenase, a T- and B-cell reactive protein that induces partial protection when co-administered with an interleukin-12-expressing plasmid in a DNA vaccine formulation**. *J Med Microbiol* 2002, **51**(8):661-671.

55.	Sanakkayala N, Sokolovska A, Gulani J, Hogenesch H, Sriranganathan N, Boyle SM, Schurig GG, Vemulapalli R: **Induction of antigen-specific Th1-type immune responses by gamma-irradiated recombinant *Brucella* *abortus* RB51**. *Clin Diagn Lab Immunol* 2005, **12**(12):1429-1436.

56.	Schurig GG, Roop RM, 2nd, Bagchi T, Boyle S, Buhrman D, Sriranganathan N: **Biological properties of RB51; a stable rough strain of *Brucella* *abortus***. *Vet Microbiol* 1991, **28**(2):171-188.

57.	Stevens MG, Olsen SC, Palmer MV, Pugh GW, Jr.: **Immune responses and resistance to brucellosis in mice vaccinated orally with *Brucella* *abortus* RB51**. *Infect Immun* 1996, **64**(11):4534-4541.

58.	Stevens MG, Olsen SC, Pugh GW, Jr., Brees D: **Comparison of immune responses and resistance to brucellosis in mice vaccinated with *Brucella* *abortus* 19 or RB51**. *Infect Immun* 1995, **63**(1):264-270.

59.	Stevens MG, Olsen SC, Pugh GW, Mayfield JE: **Role of immune responses to a GroEL heat shock protein in preventing brucellosis in mice vaccinated with *Brucella* *abortus* strain RB51**. *Comp Immunol Microbiol Infect Dis* 1997, **20**(2):147-153.

60.	Surendran N, Sriranganathan N, Lawler H, Boyle SM, Hiltbold EM, Heid B, Zimmerman K, Witonsky SG: **Efficacy of vaccination strategies against intranasal challenge with *Brucella* *abortus* in BALB/c mice**. *Vaccine* 2011, **29**(15):2749-2755.

61.	Tibor A, Jacques I, Guilloteau L, Verger JM, Grayon M, Wansard V, Letesson JJ: **Effect of P39 gene deletion in live *Brucella* vaccine strains on residual virulence and protective activity in mice**. *Infect Immun* 1998, **66**(11):5561-5564.

62.	Trant CG, Lacerda TL, Carvalho NB, Azevedo V, Rosinha GM, Salcedo SP, Gorvel JP, Oliveira SC: **The *Brucella* *abortus* phosphoglycerate kinase mutant is highly attenuated and induces protection superior to that of vaccine strain 19 in immunocompromised and immunocompetent mice**. *Infect Immun* 2010, **78**(5):2283-2291.

63.	Ugalde JE, Comerci DJ, Leguizamon MS, Ugalde RA: **Evaluation of *Brucella* *abortus* phosphoglucomutase (pgm) mutant as a new live rough-phenotype vaccine**. *Infect Immun* 2003, **71**(11):6264-6269.

64.	Velikovsky CA, Cassataro J, Giambartolomei GH, Goldbaum FA, Estein S, Bowden RA, Bruno L, Fossati CA, Spitz M: **A DNA vaccine encoding lumazine synthase from *Brucella* *abortus* induces protective immunity in BALB/c mice**. *Infect Immun* 2002, **70**(5):2507-2511.

65.	Velikovsky CA, Goldbaum FA, Cassataro J, Estein S, Bowden RA, Bruno L, Fossati CA, Giambartolomei GH: ***Brucella* lumazine synthase elicits a mixed Th1-Th2 immune response and reduces infection in mice challenged with *Brucella* *abortus* 544 independently of the adjuvant formulation used**. *Infect Immun* 2003, **71**(10):5750-5755.

66.	Vemulapalli R, Contreras A, Sanakkayala N, Sriranganathan N, Boyle SM, Schurig GG: **Enhanced efficacy of recombinant *Brucella* *abortus* RB51 vaccines against *B. melitensis* infection in mice**. *Vet Microbiol* 2004, **102**(3-4):237-245.

67.	Vemulapalli R, He Y, Boyle SM, Sriranganathan N, Schurig GG: ***Brucella* *abortus* strain RB51 as a vector for heterologous protein expression and induction of specific Th1 type immune responses**. *Infect Immun* 2000, **68**(6):3290-3296.

68.	Vemulapalli R, He Y, Buccolo LS, Boyle SM, Sriranganathan N, Schurig GG: **Complementation of *Brucella* *abortus* RB51 with a functional wboA gene results in O-antigen synthesis and enhanced vaccine efficacy but no change in rough phenotype and attenuation**. *Infect Immun* 2000, **68**(7):3927-3932.

69.	Vemulapalli R, He Y, Cravero S, Sriranganathan N, Boyle SM, Schurig GG: **Overexpression of protective antigen as a novel approach to enhance vaccine efficacy of *Brucella* *abortus* strain RB51**. *Infect Immun* 2000, **68**(6):3286-3289.

70.	Vemulapalli R, He Y, Sriranganathan N, Boyle SM, Schurig GG: ***Brucella* *abortus* RB51: enhancing vaccine efficacy and developing multivalent vaccines**. *Vet Microbiol* 2002, **90**(1-4):521-532.

71.	Wang Y, Bai Y, Qu Q, Xu J, Chen Y, Zhong Z, Qiu Y, Wang T, Du X, Wang Z *et al*: **The 16MDeltavjbR as an ideal live attenuated vaccine candidate for differentiation between *Brucella* vaccination and infection**. *Vet Microbiol* 2011, **151**(3-4):354-362.

72.	Winter AJ, Rowe GE, Duncan JR, Eis MJ, Widom J, Ganem B, Morein B: **Effectiveness of natural and synthetic complexes of porin and O polysaccharide as vaccines against *Brucella* *abortus* in mice**. *Infect Immun* 1988, **56**(11):2808-2817.

73.	Winter AJ, Schurig GG, Boyle SM, Sriranganathan N, Bevins JS, Enright FM, Elzer PH, Kopec JD: **Protection of BALB/c mice against homologous and heterologous species of *Brucella* by rough strain vaccines derived from *Brucella* *melitensis* and *Brucella* *suis* biovar 4**. *Am J Vet Res* 1996, **57**(5):677-683.

74.	Yang X, Becker T, Walters N, Pascual DW: **Deletion of znuA virulence factor attenuates *Brucella* *abortus* and confers protection against wild-type challenge**. *Infect Immun* 2006, **74**(7):3874-3879.

75.	Yu DH, Hu XD, Cai H: **A combined DNA vaccine encoding BCSP31, SOD, and L7/L12 confers high protection against *Brucella* *abortus* 2308 by inducing specific CTL responses**. *DNA Cell Biol* 2007, **26**(6):435-443.

76.	Yu DH, Li M, Hu XD, Cai H: **A combined DNA vaccine enhances protective immunity against Mycobacterium tuberculosis and *Brucella* *abortus* in the presence of an IL-12 expression vector**. *Vaccine* 2007, **25**(37-38):6744-6754.
